# Immunohistochemical Expression of DAPK-1 in Oral Leukoplakia And Oral Squamous Cell Carcinoma: A Preliminary Study

**DOI:** 10.7759/cureus.79085

**Published:** 2025-02-16

**Authors:** Petros Papadopoulos, Vasileios Zisis, Dimitrios Andreadis, Konstantinos Poulopoulos, Dimitrios Parlitsis, Konstantinos Paraskevopoulos, Pinelopi A Anastasiadou, Eleftherios Anagnostou, Konstantinos Vahtsevanos, Athanasios Poulopoulos

**Affiliations:** 1 Oral Medicine/ Pathology, Aristotle University of Thessaloniki, Thessaloniki, GRC; 2 Oral and Maxillofacial Surgery, Papanikolaou Hospital, Aristotle University of Thessaloniki, Thessaloniki, GRC; 3 Oral Medicine, Aristotle University of Thessaloniki, Thessaloniki, GRC; 4 Oral and Maxillofacial Surgery, Aristotle University of Thessaloniki, Thessaloniki, GRC

**Keywords:** cancer biomarkers, cancer stem cells, dapk-1, immunohistochemistry ihc, oral leukoplakia, oral squamous cell carcinoma

## Abstract

Introduction: The silencing of death-associated protein kinase 1 (DAPK-1) is an effective way of inactivating a tumor-suppressing mechanism. The aim of this study was to investigate the immunohistochemical expression of DAPK-1 in oral leukoplakia (OL) and oral squamous cell carcinoma (OSCC).

Methods: The immunohistochemical (IHC) detection of DAPK-1 was carried out in cases of OLs and OSCCs. DAPK-1 molecules’ tissue distribution in OLs/OSCCs tissues was evaluated using semiquantitative immunohistochemistry in representative paraffin-embedded tissue samples (57 in total) from 2004-2019, retrieved from the archives of the Department of Oral Medicine/Pathology, School of Dentistry, Aristotle University of Thessaloniki, Greece and the St Lukas Hospital of Thessaloniki, Greece. The inclusion criterion was the presence of sufficient precancerous or cancerous biological material (estimated as more than 70% per tissue specimen) in the paraffin cubes. The exclusion criterion was the opposite, i.e. the lack of sufficient material due to previous sections. Statistics for IHC were evaluated by a non-parametric Mann-Whitney U Test. A two-sided p-value < 0.05 was considered statistically significant.

Results: DAPK-1 IHC expression was increased in OLs without dysplasia and with OLs with mild dysplasia compared to moderate/severe dysplasia (p=0.019, Mann-Whitney U Test) and OSCCs (p=0.003, Mann-Whitney U Test).

Conclusions: DAPK-1 seemed to function as an oncosuppressor molecular biomarker, as its expression was decreased in areas of cellular dysplasia in OLs and in areas of OSCCs composed of less differentiated cells. The clinical application of this biomarker is that the positively stained, potentially malignant lesions are less likely to transition into malignancy, and cancerous lesions are more likely to behave non-aggressively. On the other hand, the lack of staining could signify the loss of this oncosuppressing ability, and it could be a potential prognostic biomarker for OSCC’s aggressive biologic behavior if considered with other clinical parameters and a prognostic factor of malignant transformation of potentially malignant lesions. Since this is a preliminary study, more studies with larger sample sizes are required to support these conclusions.

## Introduction

Head and neck squamous cell carcinoma (HNSCC) is a group of malignancies, involving the oral cavity, pharynx, hypopharynx, larynx, nasal cavity, and salivary glands, which together compose the seventh most common cancer diagnosis worldwide [1]. HNSCC accounts for roughly 4.5% of cancer diagnoses and deaths, with 890,000 new cases and 450,000 deaths annually [1]. A 40% rise in the incidence of HNSCC by 2040 is expected, and this is partially attributed to this condition's unpredictable clinical course and an associated pattern of late detection [2]. Although rigorous research is being conducted to achieve an early and valid diagnosis of HNSCC lesions [3-4], its measure of success, i.e., the overall survival rate, has not significantly improved over the past 20 years. International data indicate that the five-year survival rate remains below 42% [5].

Like all solid malignancies, HNSCC results from an interplay between genetic and environmental causes [6]. The evolution of HNSCC passes through a cascade of molecular as well as cytological events and pathways abandoning the idea of a single event [6-7]. In most cases, these alterations start from oral potentially malignant disorders (OPMDs) that eventually become malignancies. According to the phenotypic approach, numerous prognostic scales have been developed for oral lesions. Yet the result for HNSCC and the malignant potential of OPMDs is not determinable by available objective means [4]. This is usually observed in clinical practice because, for instance, patients with HNSCCs or OPMDs may harbor different clinical outcomes even when characterized by similar prognostic attributes, reflecting different events of genetic and epigenetic background [8-9].

Epigenetics contributes to the final characteristics of cancer, being the end point of a long multistage carcinogenic process [8-9]. In contrast with genetic changes, which are irreversible, epigenetic changes reversibly regulate gene expression on the genomic level and are regarded as an interface that could connect genotype with phenotype; hence it is relevant to the exploration of the underpinning mechanisms involved in cancer phenotypes as well as prospective therapies [8, 10]. DNA methylation has not only proved to be one of the important epigenetic marks but also the master regulator of many cellular processes, including growth and differentiation [8]. DNA methyltransferases are generally considered the key enzymes that perform and regulate the transfer of a methyl group from S-adenosylmethionine to cytosine in both forms [10]. In cancer cells, hypomethylation of DNA is linked to chromosomal instability and reduced expression of anti-tumor genes [8]. DNA hypomethylation may also contribute to genomic instability and mutagenesis [10]. On the other hand, DNA hypermethylation is responsible for the formation of cancer by inactivating tumor-suppressor genes and DNA repair genes. This is caused by the addition of a methyl group to CpG islands in the promoter regions of these genes, a process facilitated by S-adenosylmethionine, which donates the methyl group to the fifth position of the cytosine ring [11].

As far as oral cancer is concerned, epigenetic silencing is detected in many genes with tumor-suppressing abilities, a fact that undermines cellular processes such as cell cycle, apoptosis, and cellular interactions [12]. In oral cancer, more than 40 tumor suppressor genes silenced by DNA hypermethylation have been reported in a process affecting several cellular functions such as cellular cycles, apoptosis, and cell-to-cell adhesion [13-14]. On the other hand, the role of histone modifications is not limited to the inhibition of the expression of certain genes but expands to the overregulation of epithelial-mesenchymal transition, to the association with transcription that regulates processes such as anoikis and intravasation. Both these two procedures play a crucial role in metastasis [15-16]. International data denotes that the deficiency of histone modifications entails the aggressiveness of malignancy [17-18].

Aberrant DAPK DNA methylation is one of the most extensively studied events in the genesis of tumors of the head and neck. Death-associated protein kinase (DAPK) is a protein kinase with tumor-suppressing ability that among others promotes apoptosis of cells by means of INF-Ƴ implication [19]. So any epigenetic modification can alter any of DAPK's roles. More specifically, DAPK hypermethylation is able to inhibit its tumor suppressive potential, promoting in this way the rise of a malignant disorder. Such kinds of malignancies include tumors of the gastrointestinal tract as well as those of the head and neck region [19-21]. Another meta-analysis shows that the methylation of the DAPK kinase is associated with 39.7% of OSCC lesions [22]. In addition, DAPK methylation seems to be strongly associated with lymph node metastasis in patients with HNSCC [23-24]. Furthermore, a previous study by Papadopoulos et al. showed that DAPK may be expressed in the vessels underlying oral lesions as well [25].

The aim of this study was to investigate the immunohistochemical profile of DAPK-1 in oral potentially malignant disorders (OPMD) and oral cancer. In particular, our study focused on the DAPK-1 expression in oral leukoplakia (OL) and oral squamous cell carcinoma (OSCC) compared to the normal oral mucosa (NOM). The hypothesis is that DAPK-1 expression varies based on the degree of dysplasia in OL and differentiation in OSCC.

## Materials and methods

The immunohistochemical detection of DAPK-1 was carried out in cases of OLs and OSCCs. DAPK-1 molecules’ tissue distribution in OLs/OSCCs tissues was evaluated using semiquantitative immunohistochemistry in representative paraffin-embedded tissue samples retrieved from the archives of the Department of Oral Medicine/Pathology, School of Dentistry, Aristotle University of Thessaloniki, Greece and the St Lukas Hospital of Thessaloniki, Greece during the period 2004-2019. The study was conducted in accordance with the Research and Ethics Committee guidelines of Aristotle University, School of Dentistry, and the Helsinki II declaration. Approval for the present study was granted by the Ethics Committee of the School of Dentistry, Aristotle University of Thessaloniki, Greece, during its meeting on 21.11.2018, under protocol number 29/21.11.2018. The tissue specimens originate from patients who provided informed consent when they were submitted to excisional biopsy and histopathological examination. The inclusion criterion was the presence of sufficient precancerous or cancerous biological material (estimated as more than 70% per tissue specimen) in the paraffin cubes. The exclusion criterion was the opposite, the lack of sufficient material due to previous sections.

Regarding immunohistochemical analysis, 4μm-thick sections from the following tissues-samples were stained (Table 1): 30 samples of OLs, subdivided into three groups--hyperkeratosis/no dysplasia (10 samples), mild dysplasia (10 samples), and moderate to severe dysplasia (10 samples); 22 samples of OSCCs, categorized as well-differentiated (2 samples), moderately differentiated (17 samples), and poorly differentiated (3 samples); and 5 samples of normal oral mucosal tissue.

**Table 1:** Display of the epidemiological characteristics of the tissue samples. OSCC: oral squamous cell carcinoma OL: oral leukoplakia

Diagnosis	Degree of Dysplasia or Differentiation	Localization	Gender	Age
Normal		Lip	Female	79
Normal		Lip	Male	44
Normal		Buccal mucosa	Male	23
Normal		Buccal mucosa	Female	49
Normal		Tongue	Male	36
OSCC	Poorly differentiated	Alveolar process	Male	80
OSCC	Poorly differentiated	Palate	Male	35
OSCC	Poorly differentiated	Lower jaw	Male	80
OSCC	Moderately differentiated	Alveolar process	Male	62
OSCC	Moderately differentiated	Alveolar process	Female	56
OSCC	Moderately differentiated	Alveolar process	Female	59
OSCC	Moderately differentiated	Tongue	Female	67
OSCC	Moderately differentiated	Alveolar process	Female	75
OSCC	Moderately differentiated	Tongue	Female	65
OSCC	Moderately differentiated	Tongue	Female	27
OSCC	Moderately differentiated	Tongue	Female	79
OSCC	Moderately differentiated	Lip	Female	77
OSCC	Moderately differentiated	Mouth floor	Male	46
OSCC	Moderately differentiated	Alveolar process	Male	72
OSCC	Moderately differentiated	Tongue	Female	42
OSCC	Moderately differentiated	Buccal mucosa	Male	59
OSCC	Moderately differentiated	Lower jaw	Male	30
OSCC	Moderately differentiated	Tongue	Female	82
OSCC	Moderately differentiated	Gingica	Female	78
OSCC	Moderately differentiated	Gingiva	Male	57
OSCC	Well-differentiated	Buccal mucosa	Female	79
OSCC	Well-differentiated	Alveolar process	Female	72
OL	Non-dysplastic	Tongue	Male	69
OL	Non-dysplastic	Tongue	Male	53
OL	Non-dysplastic	Buccal mucosa	Female	58
OL	Non-dysplastic	Buccal mucosa	Female	55
OL	Non-dysplastic	Buccal mucosa	Female	61
OL	Non-dysplastic	Palate	Male	39
OL	Non-dysplastic	Buccal mucosa	Male	51
OL	Non-dysplastic	Gingiva	Female	60
OL	Non-dysplastic	Buccal mucosa	Female	58
OL	Non-dysplastic	Buccal mucosa	Male	55
OL	Mildly dysplastic	Buccal mucosa	Male	55
OL	Mildly dysplastic	Gingiva	Female	60
OL	Mildly dysplastic	Buccal mucosa	Female	58
OL	Mildly dysplastic	Tongue	Female	41
OL	Mildly dysplastic	Buccal mucosa	Male	51
OL	Mildly dysplastic	Buccal mucosa	Female	58
OL	Mildly dysplastic	Tongue	Male	69
OL	Mildly dysplastic	Buccal mucosa	Female	61
OL	Mildly dysplastic	Buccal mucosa	Male	56
OL	Mildly dysplastic	Buccal mucosa	Male	69
OL	Moderately or severely dysplastic	Tongue	Female	65
OL	Moderately or severely dysplastic	Tongue	Male	57
OL	Moderately or severely dysplastic	Gingiva	Female	62
OL	Moderately or severely dysplastic	Tongue	Female	70
OL	Moderately or severely dysplastic	Gingiva	Female	70
OL	Moderately or severely dysplastic	Mouth floor	Male	69
OL	Moderately or severely dysplastic	Tongue	Male	52
OL	Moderately or severely dysplastic	Tongue	Male	64
OL	Moderately or severely dysplastic	Tongue	Female	70
OL	Moderately or severely dysplastic	Tongue	Male	52

Conventional H&E staining was used to confirm the initial diagnosis for all specimens. The immunohistochemical (IHC) procedure was followed in tissue sections of NOM, OLs, and OSCC in order to detect the expression of rabbit Anti-DAPK-1 (Novus Biologicals, Centennial, Colorado, USA), diluted in 1/100.

The stepwise protocol for the immunohistochemical method included tissue fixation in 10% formalin solution, embedding in paraffin blocks, and sectioning into 4-6 μm thick slices using a Jung Biocut 2035 microtome (Rankin Biomedical Corporation, Davisburg, MI, US). The sections were applied to electrostatically charged microscope slides, deparaffinized in xylene with three changes for 5 min each, and gradually hydrated through graded alcohols-100% ethanol twice for 15 min each, followed by 90% ethanol twice for 15 min each before being washed in deionized water for 1 min with stirring. Excess liquid was aspirated from the slides, and antigen retrieval was performed by heat treatment. Endogenous peroxidase activity was blocked, and the slides were dipped in phosphate-buffered saline (PBS) solution. The primary antibody was applied for 15 min, followed by another PBS dip. EnVision (Agilent Technologies, Inc., Santa Clara, CA) was applied for 30 min, followed by another PBS dip. The DAB EnVision chromogen was then applied for a maximum of 15 min, followed by another PBS dip and a final wash in deionized water for 1 min with stirring. Hematoxylin was applied for 10 sec before the sections were mounted, covered with coverslips, and examined under a microscope and camera.

The evaluation of immunohistochemical staining was performed by microscopically examining the incisions to observe and record the results. Two observers, VZ and PP, undertook the evaluation of the immunohistochemical staining. The histochemical score was evaluated by calculating the percentage of positive cells, into a 0-3 grading scale (quantitative method) classified as: Grade 0 when less than 5% of cells were stained, Grade I when 6-25% of cells were stained, Grade II when 26-50% of cells were stained and Grade III when more than 50% of cells were stained.

The staining was deemed positive when the cytoplasm and/or the nucleus were colored brown. DAPK-1 staining was expected to be predominantly cytoplasmic. Statistical analysis was performed using SPSS version 25.0 (IBM Corporation, Armonk, NY, USA). The degree of IHC staining in OLs, OSCCs, and NOM was evaluated using the non-parametric Mann-Whitney U Test. A two-sided p-value < 0.05 was considered statistically significant.

## Results

Immunohistochemical (IHC) evaluation of the expression of DAPK-1 in OLs and OSCCs showed certain characteristics that revealed interesting details about its pattern of expression and, in some cases, confirmed its main activity as a tumor suppressor factor. Immunohistochemistry may specifically determine which cellular populations are being stained positively and thus express the DAPK-1. By defining a certain scale of staining (0-III) to designate the extent and thus the intensity of staining as a representative factor of the expression of DAPK-1 protein, it was observed that there was a significant difference in staining among normal tissue specimens and those of OLs with moderate/severe dysplasia (p=0.013, Mann-Whitney U Test) as well as between normal tissue samples and those of OSCCs (p=0.003, Mann-Whitney U Test) (normal tissue outperformed both of these conditions). More specifically, most of the normal tissue sections showed an intense staining pattern (grade III; n=4), and only one was moderately stained (grade II), whereas most OLs with moderate to severe dysplasia and OSCCs were stained faintly (grade I or II respectively). This observation was expected since DAPK-1 has a tumor-suppressing role so in lesions with malignant and pre-malignant characteristics, it is expected to be mostly inactive.

Moreover, the analysis revealed a statistically significantly higher expression of DAPK-1 in OLs with no dysplasia than in OLs with moderate/severe dysplasia (p=0.019, Mann-Whitney U Test). Also, a statistically significantly higher expression of DAPK-1 was noticed in OLs without dysplasia than in OSCCs (p=0.003, Mann-Whitney U Test). Finally, there was statistically significantly increased expression in OLs with mild dysplasia compared to OLs with moderate/severe dysplasia as well as in OLs with mild dysplasia compared to OSCCs (p=0.019 Mann-Whitney U Test and p=0.003, Mann-Whitney U Test, respectively).

Normal tissue

**Figure 1:**
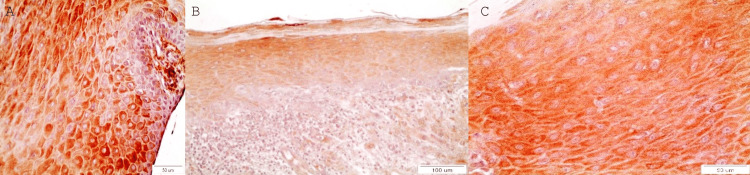
A: Overexpression of DAPK-1 in normal epithelium: cytoplasmic staining with only dispersed cells with nuclear staining (X40). B: Characteristic intense staining (Grade III) of all layers of epithelium, except the basal one in a case of OL without dysplasia (x20). C: Characteristic intense cytoplasmic expression of DAPK-1 in epithelial cells of OL without dysplasia (x40).

The expression of DAPK-1 in normal epithelial tissue adjacent to fibroma biopsies is highly intense. It is characteristic that no sample was found to be classified as Grade I staining intensity as the vast majority of them were observed to be of Grade III (Fig 1A).

OLs with no dysplasia

OLs with no dysplasia are characterized by intense expression of the DAPK-1. Indeed, 60% (6/10) of the OLs showed staining of grade III (Figure 1B-1C).

OLs with mild dysplasia

**Figure 2:**
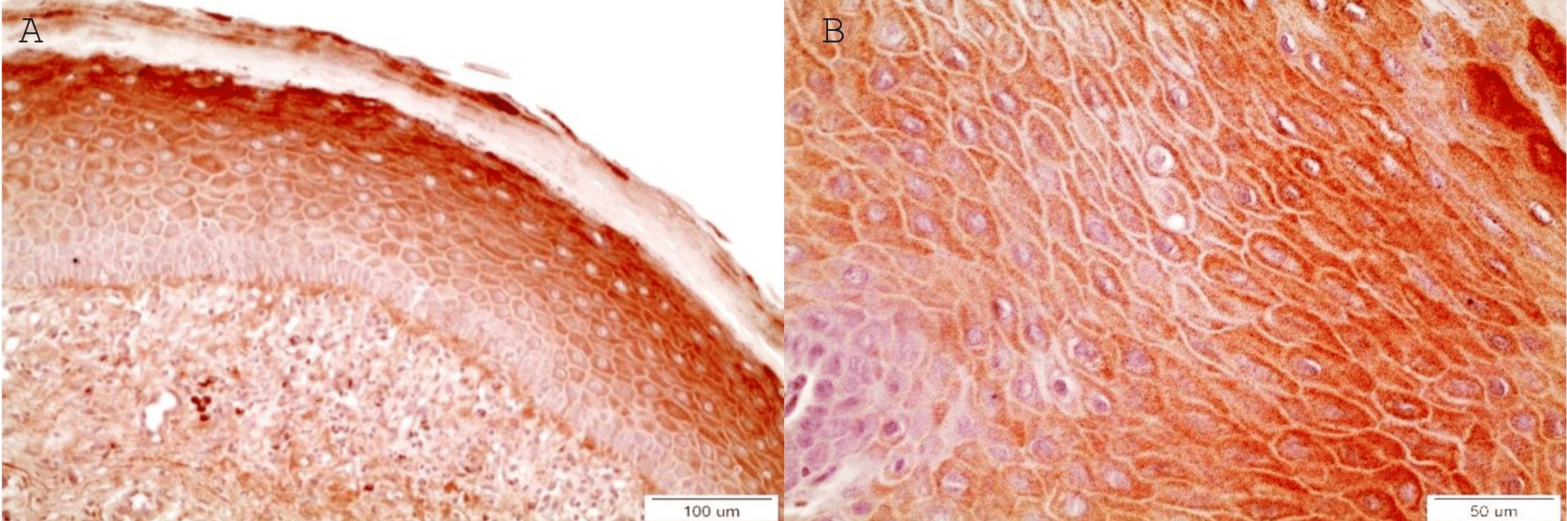
Α: Intense cytoplasmic expression of DAPK-1 in OL with mild dysplasia only in epithelial layers without dysplasia (most of spinous and granular) and absence in the lower 1/3 of ep-ithelium. Furthermore, it was observed a distinct staining in the interface between basal cells and basal membrane (x20) Β: Intense cytoplasmic staining of epithelial cells in a OL with mild dysplasia. Focal area of unstained cells among the intensively stained ones of the spinous layer (x40).

DAPK-1 expression is intense in the majority of OL lesions with mild dysplasia (Figure 2A-2B). Staining reveals a similar pattern to the one of lesions of OL with no dysplasia.

OL with moderate/severe dysplasia

**Figure 3:**
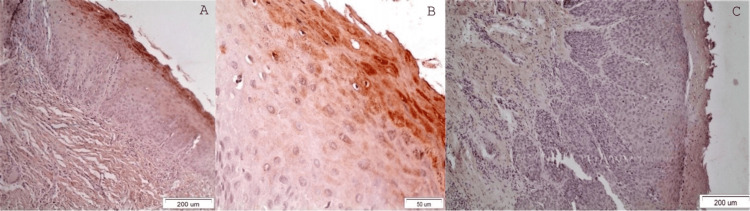
A: Positive staining of DAPK-1 is established in dispersed areas of the spinous layer and the full thickness of the granular one in a tissue section of moderate OL (X10), B: Positively stained epithelial cells in the granular layer showing nuclear as well as cytoplasmic expression of DAPK-1. Positive expression of DAPK-1 in the intercellular space of the spinous layer and few positively stained disperse cells in the upper part of it (X40). C: The expression of DAPK-1 in OL with severe dysplasia is limited to the granular layer (x10).

The vast majority of OLs with moderate/severe dysplasia seem to be positively stained in less than 50% of the epithelial cells in the upper third of the spinous and granular layers but not in the basal cell layer. In lesions with moderate dysplasia, the expression of DAPK-1 is higher than that in heavily dysplastic lesions although still weak (Figure 3A-3B). Especially in OL lesions of severe dysplasia, the expression of DAPK-1 is weak (Figure 3C).

OSCC

**Figure 4:**
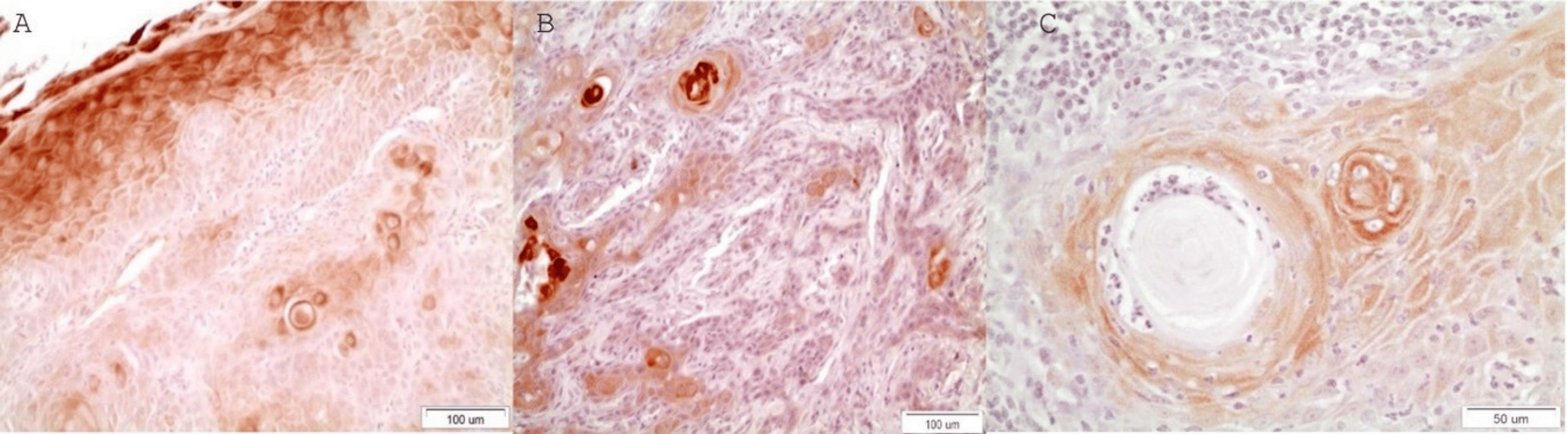
A: Dispersed epithelial islands of distinct positivity. Absence of staining in the epithelium in all layers but not the upper part of spinous and the granular layers where there is no dysplasia above neoplastic epithelial islands in a moderately differentiated case of OSCC (X20), B: Epithelial islands in a moderate differentiated OSCC case in which the more “differentiated” neoplastic cells in the center of the neoplastic islands show intense positive staining. Staining is either cytoplasmic or rarely nuclear (x20). C: Keratin pearl, in a case of well-differentiated OSCC, surrounded by DAPK-1 highly stained neoplastic epithelial cells as well as in the center of the neoplastic epithelial island. Staining is mainly cytoplasmic and, in some cases, membranous (x40).

DAPK-1 expression in the epithelial cells of the OSCCs is weak in the vast majority. DAPK-1 is highly expressed in the layers (spinous and granular) where there is no presence of dysplasia in overlying to OSCC cells’ island epithelium (Figure 4A). Moreover, in the center of neoplastic epithelial islands as well as adjacent to the keratin pearls staining is intense revealing strong DAPK-1 expression observed mostly in well and moderate grade of differentiation (Figure 4B). Staining is most of the time cytoplasmic and, on rare occasions nuclear. Very rarely membranous staining is detected (Figure 4C).

## Discussion

The present study is an integrated effort to visualize the expression pattern of DAPK-1 in OL, OSCC, and NOM with the aid of immunohistochemistry. In particular, the more intense protein expression in NOM, non-dysplastic OL, and mildly dysplastic OL aligns with the tumor-suppressing function of DAPK-1, which is expected to be expressed in these conditions of the oral epithelium. Subsequently, in moderately and severely dysplastic OL as well as in OSCC, DAPK-1 protein expression naturally regresses (fewer tissue samples manifest Grade III DAPK-1 expression), most probably due to methylation. As dysplasia worsens, and the transition to carcinoma in situ and full-fledged OSCC gradually takes place, the inactivation of tumor-suppressing genes or at least, their less intense expression constitutes a necessary prerequisite. The increasing DNA methylation of the DAPK-1 gene may not be able to affect the respective protein expression, at least not until a certain threshold is reached. The degree of dysplasia may serve as such a threshold, particularly between mild and moderate dysplasia. 

Another key finding of our study is that DAPK-1 was not expressed in the basal layer of normal epithelium. This means that kinase is not expressed in areas that contain cells with an increased proliferation capacity. On the contrary, DAPK-1 staining is intense in spinous and occasionally in granular layers in which the cells are completely differentiated. This description corresponds with the results of a similar study by Bhat et al. in 2017 that found that DAPK-1 expression was reduced in dysplastic and cancerous lesions [26]. It also found that there was an inverse correlation between DAPK-1 hypermethylation and DAPK-1 expression [26]. Nevertheless, the study of Melchers et al. found no association between DAPK-1 methylation and its protein expression [27]. This may be attributed to the fact that MSP (methylation-specific PCR) is not a quantitative method, in contrast to immunohistochemical staining analysis, which can depict the distinct cell layers and thus the number of cells being positively stained.

Bhat et al. underlined the presence of DAPK-1 in the cytoplasm of the epithelial cells, which matches the findings of the present study which indicated that staining in all kinds of epithelial cells and lesions is mainly cytoplasmic and, on rare occasions, nuclear [26], similar to a previous study by Papadopoulos et al [28]. Finally, DAPK-1 methylation might function as a trigger for carcinogenesis in oral potentially malignant lesions, receding until higher degrees of dysplasia transition to oral cancer, where methylation is expected to be present [29].

The study's limitations include the lack of tumor, nodes, and metastases (TNM) classification, the five-year survival rate of the OSCC cases, and the lack of information regarding the clinical course of OL, a potentially malignant disorder. 

## Conclusions

DAPK-1 seemed to function as an oncosuppressor molecular biomarker, as its expression was decreased in areas of cellular dysplasia in OLs and in areas of OSCCs composed of less differentiated cells. The clinical application of this biomarker is that the positively stained, potentially malignant lesions are less likely to transit into malignancy, and cancerous lesions are more likely to behave non-aggressively. On the other hand, the lack of staining may indicate the loss of this oncosuppressing ability and, when considered alongside other clinical parameters, could serve as a prognostic biomarker for OSCC's aggressive biological behavior and a predictor of malignant transformation in potentially malignant lesions. Since this is a preliminary study, more studies with larger sample sizes are required to support these conclusions.
